# Adult *Gli2^+/–^*;*Gli3^Δ699/+^* Male and Female Mice Display a Spectrum of Genital Malformation

**DOI:** 10.1371/journal.pone.0165958

**Published:** 2016-11-04

**Authors:** Fei He, Pedram Akbari, Rong Mo, Jennifer J. Zhang, Chi-Chung Hui, Peter C. Kim, Walid A. Farhat

**Affiliations:** 1 Program in Developmental & Stem Cell Biology, Research Institute, Hospital for Sick Children, Toronto, Ontario, Canada; 2 Department of Molecular Genetics, University of Toronto, Toronto, Ontario, Canada; 3 Sheikh Zayed Institute for Pediatric Surgical Innovation, Children's National Health System, Washington, DC, United States of America; 4 Division of Urology, Department of Surgery, The Hospital for Sick Children, University of Toronto, Toronto, Ontario, Canada; University of Nevada School of Medicine, UNITED STATES

## Abstract

Disorders of sexual development (DSD) encompass a broad spectrum of urogenital malformations and are amongst the most common congenital birth defects. Although key genetic factors such as the hedgehog (Hh) family have been identified, a unifying postnatally viable model displaying the spectrum of male and female urogenital malformations has not yet been reported. Since human cases are diagnosed and treated at various stages postnatally, equivalent mouse models enabling analysis at similar stages are of significant interest. Additionally, all non-Hh based genetic models investigating DSD display normal females, leaving female urogenital development largely unknown. Here, we generated compound mutant mice, *Gli2*^*+/–*^;*Gli3*^*Δ699/+*^, which exhibit a spectrum of urogenital malformations in both males and females upon birth, and also carried them well into adulthood. Analysis of embryonic day (E)18.5 and adult mice revealed shortened anogenital distance (AGD), open ventral urethral groove, incomplete fusion of scrotal sac, abnormal penile size and structure, and incomplete testicular descent with hypoplasia in male mice, whereas female mutant mice displayed reduced AGD, urinary incontinence, and a number of uterine anomalies such as vaginal duplication. Male and female fertility was also investigated via breeding cages, and it was identified that male mice were infertile while females were unable to deliver despite becoming impregnated. We propose that *Gli2*^*+/–*^;*Gli3*^Δ699/+^ mice can serve as a genetic mouse model for common DSD such as cryptorchidism, hypospadias, and incomplete fusion of the scrotal sac in males, and a spectrum of uterine and vaginal abnormalities along with urinary incontinence in females, which could prove essential in revealing new insights into their equivalent diseases in humans.

## Introduction

Disorders of sexual development (DSD) are among the most common human birth defects, affecting nearly 3% of all newborns [1]. This high incidence rate can be attributed to the complex set of developmental events that need to be harmoniously synchronized from the onset of sexual differentiation until the emergence of sexually functional adult [2]. Cryptorchidism, hypospadias, and incomplete scrotal sac fusion are the most common congenital complications in males [2, 3]. In females, disorders of sexual development typically present as congenital uterine anomalies, with particular cases such as vaginal duplication threatening child delivery [4, 5]. Despite the clinical significance of such anomalies in both males and females, there is a lack of equivalent animal models.

Male and female urogenital systems arise from the outgrowth, patterning, and differentiation of an ambisexual embryonic bulge called the genital tubercle (GT) [6, 7]. In conjunction, complete cloacal septation is required for correct urogenital and anorectal sinus formation [8–9]. Sonic hedgehog (Shh), a secreted signaling protein, is involved in GT outgrowth and patterning, as well as cloacal septation, whereas Desert hedgehog (Dhh), another hedgehog (Hh) homologue, orchestrates male gonad development and sexual differentiation [6, 8, 10]. While *Shh* null male and female mice display GT agenesis and persistent cloaca, *Dhh* null male mice are sterile, and female mice are normal [11–13]. The early and severe genital and cloacal phenotypes of *Shh* null mice and the sex specific phenotypes of *Dhh* null mice have limited the generation of a viable mouse model for common DSD affecting both male and females.

Gli transcription factors are downstream mediators of Hh signaling. Gli2 functions predominantly as an activator for Hh target genes, whereas Gli3 undergoes C-terminal truncation to form a potent repressor [14]. The balance between the two is essential for Hh signaling, as gene dosages of *Shh*, *Gli2*, and *Gli3* modulate the severity of cloacal malformations [15, 16]. Here, we report the generation of a postnatally viable genetic mouse model, *Gli2*^*+/–*^;*Gli3*^*Δ699/+*^, with reduced level of Gli2 activator and constitutive expression of Gli3 repressor (from the *Gli3*^*Δ699*^ mutant allele)[17] for studying common DSD in both male and female animals.

## Materials and Methods

The Hospital for Sick Children Research Ethics Board and The Centre for Phenogenomics have approved all animal care and use protocols used in this study. Animal euthanasia was performed via CO_2_ chambers.

### Mutant Generation and Mating

*Gli2* mutant mice carry a targeted deletion in the DNA-binding zinc-finger motif of the gene [16]. *Gli3*^*Δ699*^ mutant mice contain a targeted deletion 3’ of the zinc finger motif, rendering it a constitutively active repressor [17]. Intercrosses of *Gli2*^*+/–*^and *Gli3*^*Δ699/+*^ mice were used to generate *Gli2*^–/–^and *Gli3*^*Δ699/Δ699*^ mice, respectively. Crosses between *Gli2*^*+/–*^and *Gli3*^*Δ699/+*^ were used to generate *Gli2*^+/–^, *Gli3*^*Δ699/+*^, and *Gli2*^+/–^;*Gli3*^*Δ699/+*^ mice. All mice were of CD-1 background. Genotypes and sexual identities of the mice were determined by Polymerase Chain Reaction analysis of ear notches (postnatal 4 months) and yolk sac (E18.5) DNA using *Gli2*, *Gli3*^*Δ699*^, and *Sry* primers. All protocols were approved by the Institution’s Animal Care and Use Committee.

### Fertility Examination

To test male fertility, 12 cages were set up, each with one *Gli2*^*+/–*^;*Gli3*^*Δ699/+*^male mouse, and three normal female mice (*Gli2*^*+/–*^and *Gli3*^*Δ699/+*^). Female mice were checked for plugs every morning for three months. To test female fertility, 7 cages were set up with one normal male (*Gli2*^*+/–*^or *Gli3*^*Δ699/+*^) and 2–3 female mice per cage, with at least one *Gli2*^*+/–*^;*Gli3*^*Δ699/+*^ female. Female mice were checked for plugs every morning for three months. To test fertility of *Gli2*^*+/–*^and *Gli3*^*Δ699/+*^ mice, 5 cages, each with one male and 2–3 female mice of mixed genotypes of *Gli2*^*+/–*^, *Gli3*^*Δ699/+*^, and wild type CD-1 mice were set up. Male and female *Gli2*^*+/–*^and *Gli3*^*Δ699/+*^ mice display wild type like fertility.

### Morphological Analysis

Midday of the day of vaginal plug was considered embryonic day (E) 0.5 in embryo collection. Since *Gli2*^*–/–*^and *Gli3*^*Δ699/Δ699*^ mutant mice die at birth, and average gestation age of mice is between 19–21 days, E18.5 was chosen as the last checkpoint for prenatal genitalia development. Pregnant mice were sacrificed via CO_2_ euthanasia, and embryos were harvested, washed with PBS, and fixed in 4% paraformaldehyde overnight at 4°C for overall urogenital sinus and anogenital distance (AGD) examination under low magnification microscope, with *Gli2*^+/+^ or *Gli3*^+/+^ mice as controls. To examine the internal openness of female reproductive tract, ink was injected into the distal uterus horns, and the dark color of the ink in contrast with the pink tubules, allowed identification of tubule openness.

### Dissection, Paraffin Embedding, and Histology

Adult control mice as well as *Gli2*^+/–^;*Gli3*^*Δ699/+*^ mice were used for gross dissection of lower abdomen, and digital photographs were obtained for visual assessment of testicular location, penile/urethral size and position in males and internal genitalia in females. Pairs of control and mutant mice testes were placed side-by-side on petri dish for visual comparison of size. The longest length of each testis was measured under low power microscope and averages for both control and mutant mice were calculated.

Tissue/organs were dissected out from adult mice, and fixed in 4% paraformaldehyde overnight at 4°C. They were dehydrated, processed, and embedded in paraffin wax before sectioning at 5μm. Slides were then dewaxed, rehydrated, and stained with hematoxylin and eosin. All measurements of male external genitalia were performed according to methods described in Rodriguez et al. 2011 [18]. Seminiferous tubule count was performed via manual counting of randomized low magnification images of wild type and *Gli2*^*+/–*^;*Gli3*^*Δ699/+*^ testes stained with H&E.

### Immunofluorescence Staining

Immunofluorescence staining was performed on paraffin sections. After quenching the endogenous peroxidases with 3% H_2_O_2_ in 10% methanol, targeted antigens were retrieved by boiling the slides in an antigen-unmasking solution (H-3300, Vector Laboratories). Sections were treated with blocking reagent (all supplied by Roche) before application of primary antibodies for Sertoli and Leydig cell detection: GATA-1 (1:200, Cell Signaling Technology), and P450scc (1:100, Cell Signaling Technology), respectively. The number of Sertoli cells were quantified per seminiferous tubule by averaging the number of positive GATA-1 signals for 20 tubules within each testes (n = 5). Leydig cell signals were quantified via ImageJ software by measuring fluorescence intensities of interstitial space P450scc signals and normalized to background (n = 6). Cell proliferation was assayed by immunofluorescence staining using Ki-67 antibodies (1:100, Thermo Scientific) and apoptosis was examined using immunofluorescence staining of caspase-3 antibody (1:200, Cell Signaling Technology). Dhh expression was analyzed using Dhh antibody (1:100, Santa Cruz Biotechnology Inc.). MVH staining (1:200, ab13840) was performed in order to stain spermatogonium. All primary antibodies were incubated at 4°C overnight.

### Serum Androgen Detection

Serum from both wild type (n = 3) and *Gli2*^+/-^;*Gli3* ^Δ 699/+^ (n = 4) adult mice were extracted and sent to Aska Pharma Medical Co., LTD, Japan, where they were analyzed via liquid chromatography-tandem Mass Spectrometry (LC-MS/MS) to quantify testosterone and dihydrotestosterone.

### Statistical Analyses

All data were expressed as mean ± standard error of the mean (SEM). Possible differences in penile anatomical structure and testicular lengths, testicular weight, and seminiferous count between wild type and *Gli2*^*+/–*^;*Gli3*^*Δ699/+*^ mice were determined by Student’s two tailed t-test, and p-values<0.05 were considered significant.

## Results

### Male *Gli2*^*+/–*^;*Gli3*^*Δ699/+*^ Mice Exhibit Severely Reduced AGD and Penile Malformations

**Fig 1:**
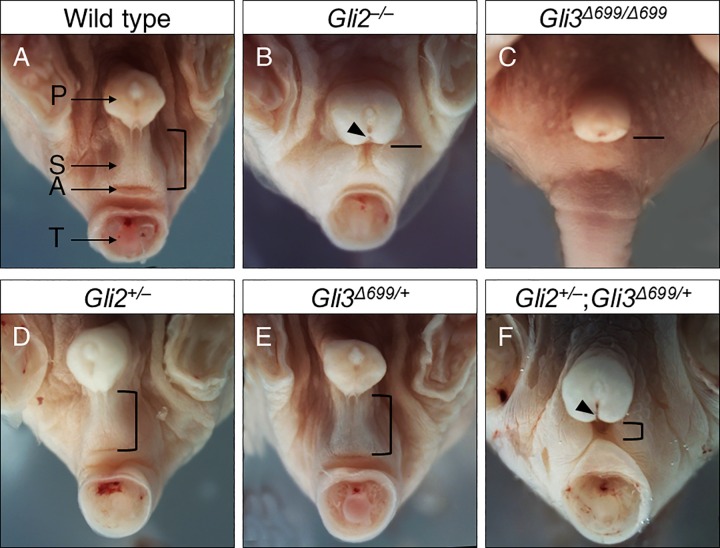
Male genital malformations in E18.5 compound mutant mice of *Gli2*^*+/–*^and *Gli3*^*Δ699/+*^. Arrows indicate major identifiable structures in the urogenital sinus area. Open bracket approximates distance between the prepuce and the anus in mice of different genotypes, with shorter bracket indicating a shortened AGD. A straight line instead indicates imperforated anus in *Gli2*^*–/–*^and *Gli3*^*Δ699/Δ699*^ mice. Black arrow indicates a ventral opening to the urethra, indicative of hypospadias. A, anus; AGD, anogenital distance; P, prepuce; S, Scrotal sac; T, tail.

Wild type male mice at E18.5 have proper prepuce development with complete fusion of scrotal sacs, while exhibiting normal AGD and urethral opening (Fig 1A, n = 8). Loss of both alleles of *Gli2* resulted in reduced AGD and incomplete fusion of the scrotum with ventrally open urethral groove in *Gli2*^–/–^mice (Fig 1B, n = 9) [16]. *Gli3*^*Δ699/ Δ699*^ mice, with two copies of constitutive repressor allele of *Gli3*, exhibited reduced AGD, a flattened scrotal sac, and imperforate anus (Fig 1C, n = 12) [17]. Loss of one copy of *Gli2* in *Gli2*^*+/‒*^ mice (Fig1D, n = 8), or gain of one copy of constitutive Gli3 repressor in *Gli3*^*+/Δ699*^ mice (Fig 1E, n = 8) did not result in any obvious phenotype [16, 17]. In contrast double heterozygous *Gli2*^*+/–*^;*Gli3*^*Δ699/+*^ mice exhibited prominent genitalia malformations, characterized by reduced AGD and incomplete fusion of the scrotum (Fig 1F, n = 15). Although it appeared that these mice displayed cloaca formation, adult stage analysis proved otherwise. In addition, all *Gli2*^+/–^;*Gli3*^*Δ699/+*^ mice displayed an open urethral groove on the ventral aspect of the developing phallus, a reliable early indication of hypospadias (Fig 1D black arrow).

**Fig 2:**
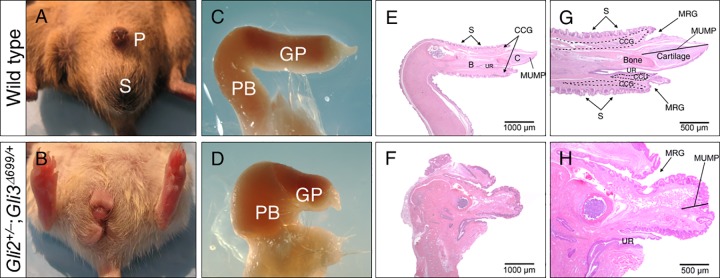
*Gli2*^*+/–*^;*Gli3*^*Δ699/+*^ adult male mice display genital malformations at 4 months of age. (A-B) Gross morphology of the external genitalia, (C-D) harvested penile structures, and (E-H) H&E mid-sagittal sections of adult penile structures highlighting the significantly reduced length of MUMP, it’s surrounding structures, MCC, and os penis. (CCG, corpus cavernosum glandis; CCU, corpus cavernosum urethrae; GP, Glans penis; GR, glanular ridge; MUMP, male urogenital mating protuberance: MCC, MUMP corpus cavernosum; MRG, MUMP ridge groove; P, Penis; PB, Penile body; S, Scrotum; UR, urethra; UM, urethral meatus).

Although male *Gli2*^+/–^;*Gli3*^*Δ699/+*^ mice lived past puberty and well into adulthood, they were not able to plug females (n = 12). To characterize these mice in their adult stages of life, we examined their genitals and gonadal phenotypes at 4 months of age. In wild type adult males, mature sexual characteristics, such as proper prepuce size and location, and completely fused scrotal sac, were apparent (Fig 2A). In contrast, all *Gli2*^+/–^;*Gli3*^*Δ699/+*^ mice displayed ambiguous or feminized appearance with reduced AGD, bifid phallus, incomplete fusion of scrotal sac, and penile malformations (Fig 2B, n = 20). Isolated penile examination revealed that the mutant phallus is reduced in length and exhibits abnormal shape at both penile body and glans penis (Fig 2C and 2D, n = 12). Histological and morphometric evaluation revealed a reduction in length of several penile features including male urethral mating protuberance (MUMP) and os penis in *Gli2*^+/–^;*Gli3*^*Δ699/+*^ mice (Fig 2E, 2F, 2G and 2H, and Table 1). In addition, structures visible dorsally to MUMP, including MUMP ridge groove (MRG), corpus cavernosum glandis (CCG), and surrounding keratinized spines, were disorganized and severely shortened in *Gli2*^+/–^;*Gli3*^*Δ699/+*^male mice (Fig 2G and 2H). As a consequence, mutant mice also displayed shortened urethra (UR)(Fig 2G and 2H) [18].

**Table 1:** Morphometric data of wild type and *Gli2*^*+/–*^;*Gli3*^*Δ699/+*^ penile structures.

Feature	Wild type (μm)	*Gli2*^*+/–*^;*Gli3*^*Δ699/+*^ (μm)
**MUMP Length**	1670 ± 170	564 ± 90 *
**Distance (MUMP tip to urethral meatus)**	930 ± 80	318 ± 60 *
**MCC length**	560 ± 70	250 ± 50 *
**Bone Length**	3530 ± 320	678 ± 120 *
**Organ Width**	1980 ± 210	843 ± 260 *

Data represented as SEM

* Significantly different from wild type (P<0.05).

n = 8 for wild type, n = 12 for *Gli2*^*+/–*^;*Gli3*^*Δ699/+*^

### Male Gli2+/–;Gli3Δ699/+ Mice Exhibit Undescended, Hypoplastic Testes

**Fig 3:**
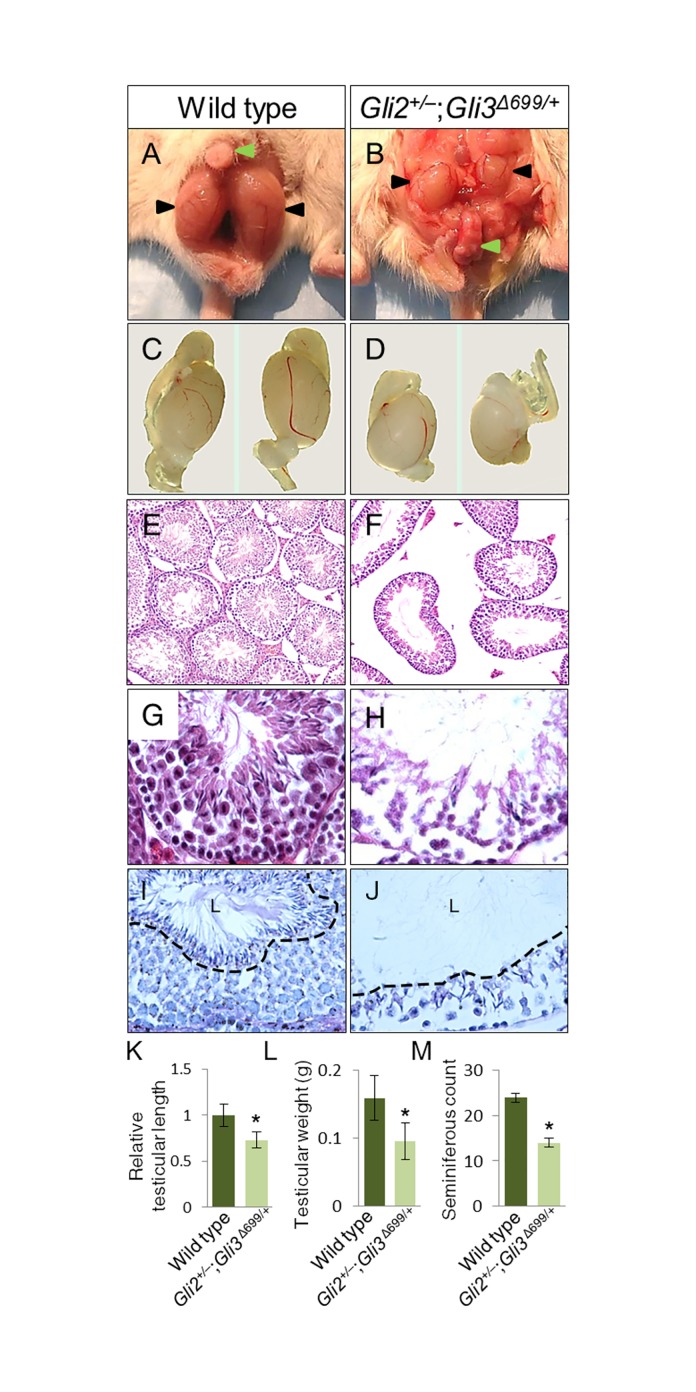
Adult *Gli2*^*+/–*^;*Gli3* ^*Δ699/+*^ male mice display undescended, hypoplastic testes, and sperm maturation arrest. (A-B) Gross dissection of the lower abdomen and genital region. (C-D) Isolated testes comparison of 4 months old wild type and *Gli2*^*+/–*^;*Gli3*^*Δ699/+*^ mutant, respectively. (E-F) H&E staining of testes sections under 10X magnification. (G-H) 40X magnification. (I-J) PAS staining of seminiferous tubules, black dashed line separating luminal space labeled “L”. (K-M) A comparison between wild type and *Gli2*^*+/–*^;*Gli3*^*Δ699/+*^ testicular size (n = 20, P<0.05) and weight (n = 20, P<0.05) respectively. (K) Relative seminiferous tubule count among wild type and *Gli2*^*+/–*^;*Gli3*^*Δ699/+*^mice (n = 20, P<0.05).

Next, we examined the gonads of *Gli2*^*+/–*^;*Gli3*^*Δ699/+*^ adult male mice. In wild type mice testes lay below the phallus level in their proper scrotal sac position (Fig 3A, n = 8). 80% of *Gli2*^*+/-*^;*Gli3*^*Δ699/+*^ mice showed varying degrees of incomplete testicular descent, of which 70% were bilaterally, and 30% were unilaterally undescended testes (Fig 3B, n = 20). It was readily apparent that testicular size was reduced in *Gli2*^*+/–*^;*Gli3*^*Δ699/+*^(Fig 3C and 3D, n = 20). Histological analyses revealed that *Gli2*^*+/–*^;*Gli3*^*Δ699/+*^ testes were hypoplastic, with enlarged seminiferous tubule luminal space, expanded interstitial space, reduced cellular density, and detached interstitial cells from the basal membrane (Fig 3E–3H, n = 20). PAS staining revealed maturation arrest as no mature spermatozoa were observed in the luminal space of *Gli2*^*+/–*^;*Gli3*^*Δ699/+*^, indicated via black dashed line (Fig 3I and 3J). Morphometric analysis revealed that average testicular length was reduced by 27% in *Gli2*^*+/–*^;*Gli3*^*Δ699/+*^ mice (0.866 cm vs. 0.636 cm, n = 20, P<0.05) (Fig 3C, 3D, and 3K) and average testicular weight was reduced by 40% (0.159g vs. 0.095g, n = 20, P<0.05) (Fig 3L). *Gli2*^*+/–*^;*Gli3*^*Δ699/+*^ mice had a 43% reduction of seminiferous tubule count compared to wild type testes (Fig 3M; n = 20, P<0.05). Analysis of epididymis, seminal vesicle, and prostate also revealed reduced size in *Gli2*^*+/–*^;*Gli3*^*Δ699/+*^ mice (data not shown).

**Fig 4:**
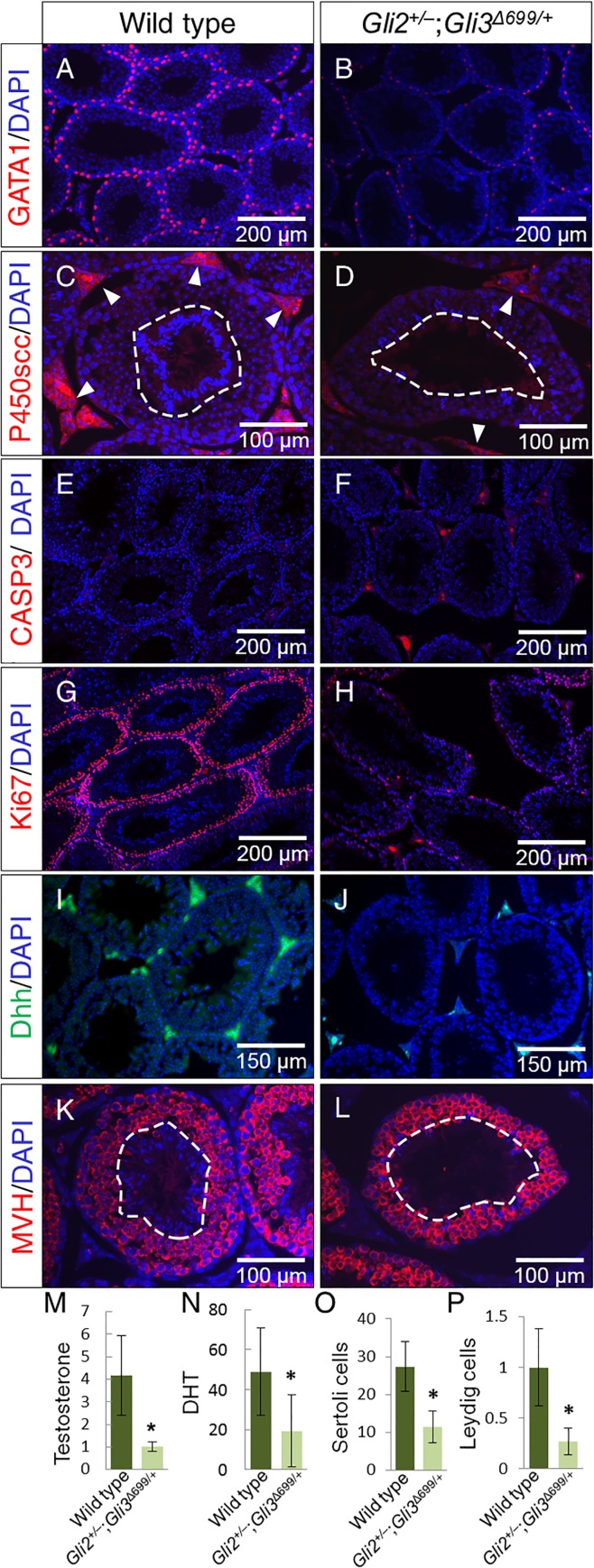
Marker analysis of wild type and *Gli2*^*+/–*^;*Gli3*^*Δ699/+*^ testes. Analysis of testicular specific Sertoli (A-B) and Leydig (C-D) cells responsible for sperm maturation and testosterone production respectively via GATA1 and P450scc immunofluorescence staining. (E-F) Caspase-3 was used as an indicator of apoptosis whereas Ki67 (G-H) was used as a proliferation marker. (I-J) Dhh staining was performed to analyze levels of Desert hedgehog within adult testes. (K-M) MVH staining for spermatogonia. Quantification of serum (M) testosterone (ng/ml) and (N) dihydrotestosterone (pg/ml) (Wild type (n = 3), *Gli2*^*+/–*^;*Gli3*^*Δ699/+*^ (n = 4), p<0.05). (O) Average Sertoli cell count per seminiferous tubule, (P) Relative fluorescence intensity of interstitial spaces of testes.

To gain insight into spermatogenesis in *Gli2*^*+/–*^;*Gli3*^*Δ699/+*^ mice, Sertoli and Leydig cell populations were analyzed via GATA1 and P450scc immunofluorescence labeling, respectively. GATA1 staining revealed a reduction of Sertoli cells, the cells responsible for Dhh production in the testes (Fig 4A and 4B). P450scc staining revealed a reduction of Leydig cells in the mutant testes (Fig 4C and 4D), and strong DAPI signals from the seminiferous tubule lumen, where mature spermatozoa reside (outlined with white dashed circle in Fig 4C and 4D). Increased Caspase-3 labeling in the interstitial space (Fig 4E and 4F) revealed increased apoptosis. Ki-67 labeling revealed significantly reduced proliferation (Fig 4G and 4H) along the basal seminiferous tubules in *Gli2*^*+/–*^;*Gli3*^*Δ699/+*^ testes. Consequently, Dhh, the main mediator of testicular development, was also significantly reduced in the mutant testes (Fig 4I and 4J). MVH staining revealed the presence of spermatogonia in *Gli2*^*+/–*^;*Gli3*^*Δ699/+*^ testes, however, no DAPI staining corresponding to mature spermatozoa were observed in the luminal space outlined by white dashed circle (Fig 3K and 3L and Fig 4C and 4D). Serum androgen levels were analyzed, and reduced amounts of testosterone and dihydrotestosterone were found in *Gli2*^*+/–*^;*Gli3*^*Δ699/+*^mice (Fig 3M and 3N, n = 4) with respect to control mice (n = 3). Quantification of Sertoli and Leydig cells indicated significantly reduced respective cell signals (Fig 4O and 4P).

### Female *Gli2*^*+/–*^;*Gli3*^*Δ699/+*^ Mice Display Reduced AGD, Uterine Abnormalities, and Urinary Incontinence

**Fig 5:**
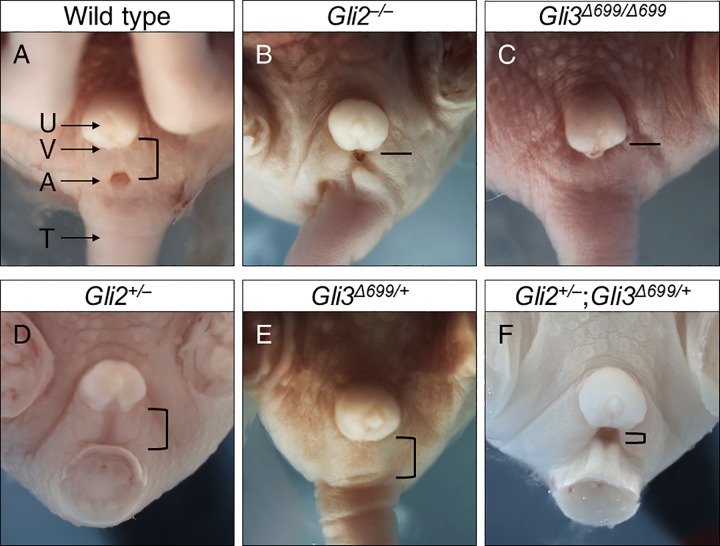
E18.5 female *Gli2*^*+/–*^;*Gli3*^*Δ699/+*^ mice display abnormal urogenitalia. Arrows indicate major structures within the urogenitalia region of female mice. AGD is indicated via the size of an open brackets measuring distance between the vagina and the anus. Straight line indicates imperforate anus in *Gli2*^*–/–*^and in *Gli3*^*Δ699/Δ699*^ respectively. (A, anus; T, tail; U, urethra; V, vagina).

**Fig 6:**
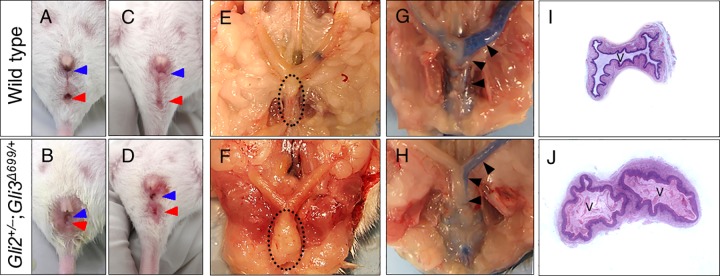
Internal vaginal and uterine phenotype of adult female *Gli2*^*+/–*^;*Gli3*^*Δ699/+*^ mice. (A-D) Gross external morphology of urogenitalia sinus in female wild type and *Gli2*^*+/–*^;*Gli3*^*Δ699/+*^ mice, blue arrows indicate vaginal opening whereas red arrows indicate anus, and their separation indicates a non-cloacal opening. (E-F) Characteristics of internal reproductive structures, dotted oval indicates the vaginal tube, enlarged in *Gli2*^*+/–*^;*Gli3*^*Δ699/+*^ mice due to double vagina and hypertrophy. (G-H) ink injection assays highlighting the extent of uterine obstruction. (I-J) H&E sectioning of genitalia indicating double vaginas in 30% of *Gli2*^*+/–*^;*Gli3*^*Δ699/+*^ mice (n = 14).

E18.5 wild type females display a well separated vagina and urethra (Fig 5A, n = 8). In contrast, both female *Gli2*^–/–^and *Gli3* ^*Δ699/ Δ699*^ mice displayed imperforate anus and were not viable (Fig 5B and 5C, n = 6 and n = 8, respectively) [16, 17]. *Gli2*^+/–^and *Gli3*^Δ699/+^ female mice exhibited normal external genitalia phenotype (Fig 5D and 5E, n = 6 and n = 8 respectively)[16]. *Gli2*^*+/–*^;*Gli3* ^*Δ699/+*^ female mice showed affected urogenital sinus with reduced AGD (Fig 5F, n = 11). However, unlike *Gli2*^–/–^and *Gli3* ^*Δ699/Δ699*^ mice, they survived postnatally. Adult female *Gli2*^*+/–*^;*Gli3*^*Δ699/+*^ mice displayed enlarged clitoris and vaginal openings (Fig 6A–6D, n = 10). Although AGD was severely reduced, it was identified that the urethra, vagina, and anus all remain separate in these mice (Fig 6B and 6D), similar to males. Anatomical and histological evaluations along with dye injection assays revealed vaginal wall hypertrophy in 30% (Fig 6E and 6F), uterine stenosis in 40% (Fig 6G and 6H), and double vaginas in 30% of female mice (Fig 6I and 6J, n = 14). As such, although female *Gli2*^+/–^;*Gli3*^*Δ699/+*^ mice were able to become impregnated by wild type males, they were unable to deliver the pups (n = 7). In addition, female *Gli2* ^+/–^;*Gli3*^*Δ699/+*^ mice invariably displayed urinary incontinence (data not shown).

## Discussion

Disorders of sexual development cover a spectrum of pathologies ranging from slight genital malformations to complete sex reversals in males and females [1]. Hh signaling has been identified as a key regulator of processes that are vital to proper genital development such as GT outgrowth and patterning, onset of sexual differentiation, and cloacal septation [8, 16]. As such, mice with mutations in the Hh signaling pathway display GT agenesis, imperforate anus, or persistent cloaca, phenotypes too severe for postnatal viability [7, 12– 14]. Although Gli transcription factors have been previously shown to regulate the anatomical sexual dimorphism of the GT, a model with a precise level of Hh signaling low enough to display the spectrum of urogenital malformations, and high enough to maintain viability, has not yet been reported [13, 15, 16, 19]. This is of particular significance, because human equivalent cases are diagnosed and treated postnatally in practice [20,21].

Cryptorchidism, hypospadias, and incomplete scrotal sac fusion are often linked congenital reproductive anomalies in male newborns [3]. To our knowledge, the only viable mouse model which exhibits these three phenotypes is the androgen receptor knockout (ARKO) mouse model [22]. However, although ARKO mice have expanded our understanding of these disorders, their phenotypes including 80% testes size reduction, and agenesis of vas deferens, epididymis, seminal vesicle, and prostate are rather extreme [22]. Here, our *Gli2*^*+/–*^;*Gli3* ^*Δ699/+*^ mouse model displays less severe phenotypes by presenting only a 40% reduction of testes size, with moderate reduction of the epididymis, seminal vesicle, and prostate. As such, *Gli2*^*+/–*^;*Gli3* ^*Δ699/+*^ mice can be used as a relevant animal model for studying DSD.

Male *Gli2*^*+/–*^;*Gli3* ^*Δ699/+*^ mice displayed several genital and gonadal defects, which could all contribute to infertility. However, due to their inability to plug females, the primary reason for their infertility here is attributed to structural malformations of their penis. Of significance, the *Gli2*^*+/–*^;*Gli3*^*Δ699/+*^ phallus displayed reduced length of MUMP and OS penis, two structures that physically facilitate mating [18]. In addition, male *Gli2*^*+/–*^*;Gli3*^*Δ699/+*^ testes displayed significantly reduced seminiferous tubule count with increased interstitial and luminal space. The luminal space of wild type testes displayed a wispy texture, indicative of spermatozoa, which is absent in the luminal space of *Gli2*^*+/–*^;*Gli3*^*Δ699/+*^ testes, suggesting defective spermatogenesis in the mutants [22]. This is supported by both Sertoli and Leydig cell count reductions in the mutant testes. The reduction of Leydig cells, as well as the similarities shared between *Gli2*^*+/–*^;*Gli3*^*Δ699/+*^ and ARKO male mice suggest compromised androgen production in our model. As such we analyzed serum androgen levels, and indeed *Gli2*^*+/–*^;*Gli3*^*Δ699/+*^ mice display reduced testosterone and dihydrotestosterone. This reduction rather than its abolishment as seen in ARKO mice may be the underlying reason behind the milder phenotypes observed.

ARKO mice as well as *Dhh*, *Insl3*, and *Fkbp52* null mice, which are popular models of male genitalia and/or gonadal malformations, all display normal female counterparts [10, 22–24]. In contrast, *Shh* null mice, which display severe cases of persistent cloaca, and *Gli2* as well as *Gli3* null mice, which display imperforate anus with recto-urethral fistula and anal stenosis respectively, reveal the importance of hedgehog signaling in anorectal and urogenital development in both male and female [16]. Here, for the first time, we report a viable mouse model, which displays urogenital sinus anomalies, vaginal duplication, and urinary incontinence in its females. All *Gli2*^+/–^;*Gli3* ^Δ699/+^ females displayed uterine abnormalities, with a variety of anatomical disturbances. Although the reason for their inability to successfully deliver pups is not clearly known, internal uterine and vaginal stenosis, vaginal wall hypertrophy, and vaginal duplication may be significant contributing factors. This speculation rests on the results of the dye injection experiments but ongoing tests are currently being performed to elucidate this further. It has been previously shown that Gli2 regulates bladder mesenchymal patterning, which is required for proper smooth muscle formation and ultimately functioning of the bladder [25]. In our *Gli2*^+/–^;*Gli3* ^Δ699/+^ model, reduced Gli2 levels as well as the constitutive expression of Gli3 repressor could potentially affect mesenchymal patterning within the bladder, resulting in defective bladder wall formation and maturation, ultimately leading to urinary incontinence in adult female mice. Importantly, this urinary bladder anomaly is quite representative of those observed in humans as urinary incontinence is significantly more prevalent in females with cloacal anomalies,anorectal malformation, or urogenital sinus anomalies [26, 27]. Thus, the histological infrastructure and functional characteristics of the urinary bladders in *Gli2*^+/–^;*Gli3* ^Δ699/+^ female mice warrant further investigation.

## Conclusions

Proper levels of Hh signaling are required for normal development. While drastic reduction or complete inactivation of the Hh pathway leads to severe hindgut malformations and embryonic lethality, moderate reduction of Hh pathway activity results in viable mice with urogenital malformations and compromised sexual differentiation. Here, we show that a precisely reduced level of Hh pathway activity in *Gli2*^*+/–*^;*Gli3*^*Δ699/+*^ mice generates a spectrum of urogenital malformations with reduced sexual differentiation in both male and female animals. Importantly, the anomalies observed in these mice strongly resemble those of common human clinical cases of cryptorchidism, hypospadias, and incomplete fusion of the scrotal sac in males, and various cases of genital and uterine abnormalities such as vaginal duplication and urinary incontinence in females. Thus, *Gli2*^*+/–*^;*Gli3*^*Δ699/+*^ mice could serve as excellent animal models for studying the pathogenesis of urogenital malformations for insight into their equivalent diseases in humans.
